# ESAT-6/CFP10 Skin Test Predicts Disease in *M. tuberculosis*-Infected Guinea Pigs

**DOI:** 10.1371/journal.pone.0001978

**Published:** 2008-04-23

**Authors:** Karin Weldingh, Peter Andersen

**Affiliations:** Department of Infectious Disease Immunology, Statens Serum Institut, Copenhagen, Denmark; McGill University, Canada

## Abstract

**Background:**

Targeted preventive chemotherapy of individuals with progressive subclinical (incipient) disease before it becomes contagious would break the chain of tuberculosis transmission in high endemic regions. We have studied the ability of a skin test response to ESAT-6 and CFP10 (E6/C10) to predict later development of tuberculosis disease in the guinea pig model.

**Methods and Findings:**

Guinea pigs, either vaccinated with BCG or unvaccinated, were infected with a low dose of *Mycobacterium tuberculosis* by the aerosol route and the development of delayed type hypersensitivity responses to E6/C10 and to purified protein derivative (PPD) were followed until the onset of clinical disease. We demonstrated a negative correlation between the size of the skin test response and the time to the onset of clinical disease; a large E6/C10 skin test response correlated to a shorter survival time post skin testing, while a small E6/C10 skin test reaction correlated with a longer survival time (*r* = −0.6 and P<0.0001). No correlation was found using PPD.

**Conclusions:**

Our data suggest that it may be possible to develop a prognostic skin test based on E6/C10 that will allow the identification of individuals with incipient disease, who have the highest risk of developing active tuberculosis in the near future.

## Introduction

The global tuberculosis (TB) epidemic is continuously fuelled by transmission of *Mycobacterium tuberculosis* (MTB) before the patient is diagnosed [1] This problem is exacerbated by the unpredictable emergence of new cases arising from the enormous pool of latent TB. The specific detection of latent *M. tuberculosis* infection (LTBI) has recently become possible with the new blood assays; the ELISA based assay QuantiFERON-TB Gold (Cellestis, Australia) and the ELISPOT based assay T-SPOT.TB (Oxford Immunotec, United Kingdom). These interferon gamma (IFN-γ) release assays (also known as IGRAs) are based on measurement of IFN-γ release from peripheral blood cells after stimulation with the MTB specific antigens; early secreted antigen target 6 (ESAT-6) and culture filtrate protein 10 (CFP10) [2]. In developed countries these IGRAs are being used as tools for contact tracing and to support the decision to initiate preventive therapy for LTBI as they have proved more specific and informative than the Tuberculin skin test (TST) [3]. The priorities in high endemic regions are, however, very different and emphasis is often placed on identification and chemotherapy of patients with active TB, as these cases are already infectious and thus the major source of transmission and new cases. Furthermore, the immense numbers of latently-infected individuals in these regions makes identification and treatment of all latent infections virtually impossible with existing resources. On a global level, preventative therapy is recommended by the World Health Organization (WHO) only for HIV-infected persons who have a coincident latent tuberculosis infection or who are at high risk of exposure e.g household contacts of patients with confirmed tuberculosis [4]. However, the development of a prognostic marker for TB would allow treatment of the small fraction of latently infected individuals in high endemic regions most at risk of developing contagious TB. By initiating preventive treatment only for those with a high risk of incipient disease, breaking the chain of MTB transmission becomes a realistic prospect, which would contribute greatly to global TB control efforts.

Expression and secretion of ESAT-6 and CFP10 are tightly linked to the growth of *M. tuberculosis* and there is increasing evidence that cell mediated immune (CMI) responses to ESAT-6 correlate closely with bacterial replication in animal models, both with regard to bacterial numbers and with regard to the development of pathology [5], [6]. Furthermore, a small study in recently-exposed, healthy contacts reported an association between a strong IFN-γ response to ESAT-6 and the risk of developing TB in the 1–2 years after exposure when compared to exposed contacts with low ESAT-6 responses [7].

In the present study we hypothesized that a skin test reaction induced by a combination of ESAT-6 and CFP10 (E6/C10) could be used as a marker for incipient disease in the highly sensitive guinea pig model. Bacille Calmette-Guérin (BCG) vaccination prolongs the lifespan of MTB infected guinea pigs and this model has therefore been used extensively for vaccine testing purposes where weight loss is used as a sensitive parameter for the development of active disease [8], [9]. In addition to vaccine evaluation, the guinea pig model has been widely used for the development, testing and standardization of skin test reagents such as purified protein derivative (PPD). The use of ESAT-6 and CFP10 as skin test reagents have already been described in this model, in which a combination of these two molecules proved a specific and sensitive reagent for the detection of TB infection [10].

We demonstrate that in a group of BCG vaccinated and unvaccinated guinea pigs with a wide range of lifespan post TB infection, the size of the skin test reaction to E6/C10 (but not to PPD) early after infection, predicts the time period before development of clinical disease. Our data suggest that it would be possible to develop a prognostic skin test based on these specific antigens; and that individuals with the highest risk of later developing active TB could be identified based on the size of their ESAT6/CFP10 skin test. Combined with targeted chemotherapy, this approach offers the possibility of breaking the cycle of MTB transmission by treating high-risk individuals before they become infectious.

## Materials and Methods

### Antigens

ESAT-6 and CFP10 were produced as HIS-tagged recombinant proteins in *E. coli* as described previously. Briefly, the genes were cloned into the pMCT6 expression vector and the proteins purified in the presence of 8 M urea by metal ion affinity chromatography on a Ni+ column followed by an anion exchange column [11], [12]. The endotoxin levels for both of the proteins were <50 EU/mg.

### Animals

Outbred female Dunkin Hartley guinea pigs (weighing 250–300 g) were purchased from Harlan Scandinavia (Allerød, Denmark). After infection, the animals were housed in cages contained within laminar flow safety enclosures in a biosafety Level III facility and provided with irradiated food and filtered drinking water. The use of guinea pigs was conducted in accordance with the regulations set forward by the Danish Ministry of Justice and the animal protection committees and in compliance with European Community Directive 86/609 and the U.S Association for Laboratory Animal Care recommendations for the care and use of laboratory animals.

### Experimental infection and vaccination

Vaccinations were performed by subcutaneous injection of 0.5 ml (5×10e4 CFU) *Mycobacterium bovis* BCG (strain Danish 1331, Statens Serum Institut, Copenhagen, Denmark). The animals were infected with *M. tuberculosis* eight weeks after BCG vaccination.

*M. tuberculosis* strain Erdman was grown in 37°C on modified Sauton medium enriched with 0.5% sodium pyruvate and 0.5% Glycose. The animals were infected by the aerosol route with 10–20 CFU *M. tuberculosis* strain Erdman using a Gas-Col ™ nebuliser [13]. The development of disease was followed by weighing the guinea pigs once to twice a week. Each animal was euthanized, according to animal care guidelines, when it had lost 15% of body weight compared to the maximum weight or if other signs of severe illness were observed such as fatigue, breathing problems or anorexia.

### Skin testing

Guinea pigs were divided into groups of five to seven animals and tested by intradermal injection on the flank with 0.1 ml of reagent diluted in endotoxin-free phosphate buffered saline (PBS). All animals were tested with PPD, E6/C10, ESAT-6, CFP10 and PBS. The locations of the skin tests were randomised and the diameters of the erythema were read after 24 hours in a blinded manner by two independent readers. The variation between the two readings was always less than 10% and the results were expressed as the mean of the two readings. A mean reaction diameter of 5 mm was regarded as positive. The PPD RT23 (Statens Serum Institut, Denmark) was used at a dose of 10 T.U (1 T.U = 0.02 ug). The E6/C10 was used at a concentration of 1ug ESAT-6+1 ug CFP10 mixed together in 0.1 ml PBS.

Pilot experiments in uninfected animals revealed that sensitization by E6/C10 skin-testing can occur if the animals are repeatedly skin-tested with time interval between injections of 4 weeks or less (Weldingh, unpublished data). To avoid sensitization, the experiments were planned so that repeated injections were administered with more than 8 weeks interval and a maximum of 4 tests were performed for each animal. With this schedule, no sensitization was observed in the control animals (data not shown). The skin testing was performed at the following pre-determined intervals post infection: week 4, 8, 12, 20, 35, and 52. In order to allow for the resting period of 8 weeks, groups were either tested at week 4, 12, 35 and 52; week 8, 20, 35 and or week 12, 20, 35, 52 post infection. To avoid the negative influence of skin test anergy often found in advanced stages of disease [14] we excluded animals which had already starting losing weight (defined as a weight loss of >50 g over a three week period) before the skin test was performed.

### Statistical methods

For comparison of Kaplan-Meier survival curves, medium survival time and calculation of hazard ratio the software Prism 4.0 from GraphPad was used. The hazard ratio was calculated using a logrank test (in some places referred to as the Mantel-Haenszel logrank test). The non-parametric Spearman correlation analysis was performed by the Bio-statistics department at Statens Serum Institut using SAS version 9.1. In order to perform a nonparametric Spearman correlation analysis only one (the first) data point from each animal was included. The correlation coefficient *r* was computed using Fisher's z transformation. A P value less than 0.05 was considered significant.

## Results

### Skin test responses in vaccinated versus non-immunized guinea pigs

Guinea pigs are highly susceptible to TB but a BCG vaccination prior to infection can delay the onset of disease by approximately 6–7 weeks. After an aerosol infection with MTB, the number of bacteria in the lungs of BCG vaccinated animals is approximately 20–30 times lower than in unvaccinated animals measured at week 7 and 13 after infection (data not shown). Using this animal model we monitored and compared the development of skin test responses from *M. tuberculosis* challenge to the onset of disease in susceptible (non BCG vaccinated) and partly protected animals (BCG vaccinated). Weight loss was used as an objective non-invasive parameter for onset of clinical disease and the guinea pigs were euthanized after a loss of 15% of their maximum weight (data not shown). In addition, the guinea pigs were observed daily for sign of disease such as anorexia, fatigue and breathing problems and if any abnormalities were observed, the animals were euthanized.

**Figure 1:**
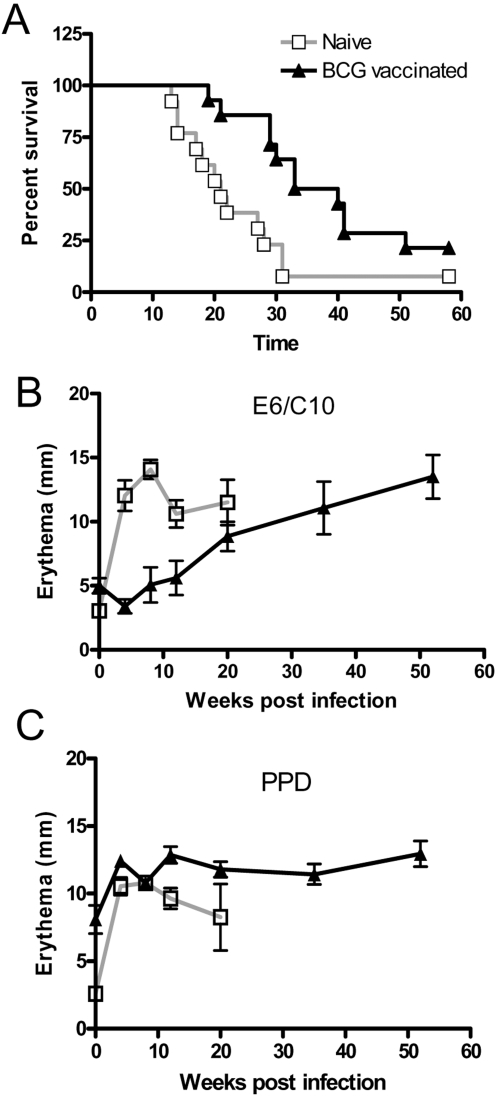
Survival curves and skintest responses in *M. tuberculosis* infected guinea pigs. Survival curves of BCG vaccinated guinea pigs (triangles) and unvaccinated guinea pigs (open squares) after *M. tuberculosis* infection. The animals were sacrificed when they had lost 15% of the maximum weight or had clinical symptoms indicative of severe TB (Fig 1A). Mean skin test reaction (+/−SEM) for *M. tuberculosis-*infected unimmunized and BCG vaccinated guinea pigs (n = 7), measured as erythema after intradermal injection of 2 ug E6/C10 (Fig. 1B) or 10 T.U. PPD (Fig. 1C). Each animal received a maximum of 4 tests with more than 8 weeks interval and this schedule did not sensitize naïve animals. The experiment was repeated twice with the same overall result and data shown for a representative experiment.

Survival curves from the BCG vaccinated (n = 14) and the unvaccinated (n = 14) groups of guinea pigs are shown in figure 1A. The first naïve un-vaccinated animal was euthanized 13 weeks after infection and 93% (13 out of 14) of the animals in this group were euthanized by week 31. The development of disease was delayed in the BCG vaccinated animals-in this group, the first animal was euthanized at week 19. The median survival time for the non-vaccinated group was 21 weeks compared to 36.5 weeks for the BCG vaccinated animals and the survival curves for the naïve and the BCG vaccinated animals were significantly different (log rank test; p = 0.0068). At termination of the experiment at week 58 post infection, four animals in the BCG vaccinated group and one animal in the naïve group were still alive.

The delayed disease development in the BCG vaccinated animals allowed evaluation of skin reactions in different stages of the infection and the E6/C10 and PPD induced skin test reaction was followed at various time points after infection (figure 1, B+C). For longitudinal evaluation of the development of skin test reactions a maximum of 4 tests with more than 8 weeks interval were performed for each animal and with this schedule, no sensitization was observed in the control animals (data not shown). After BCG vaccination and prior to *M. tuberculosis* infection all of the tested (n = 7) BCG vaccinated animals were positive to a PPD skin test (mean of 8 mm), while none of the naïve animals were PPD positive at this time point. As expected, neither BCG vaccinated nor naive animals responded to the E6/C10 skin test prior to infection, using a 5 mm cut-off.

**Figure 2:**
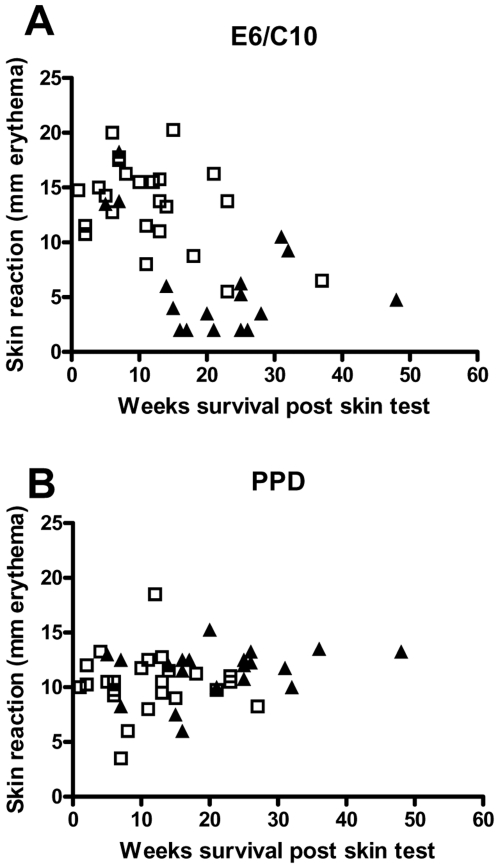
Relationship between disease outcome and skin test response to E6/C10 and PPD. Depicted is the size of the skin test reaction to E6/C10 (figure A) and PPD (figure B) against weeks of survival post skin testing for each animal. Each symbol represents a skin reaction from one animal tested either at week at 4, 8 or 12 weeks post infection (the first skin test for each animal). There is a negative correlation between E6/C10 skin test response and survival time post skin testing (P<0.0001) but no correlation between PPD responses and survival (P = 0.095). Triangles represent data points from BCG vaccinated animals and open squares represent data points from naïve animals.

Four weeks after infection all animals tested (n = 14) gave a positive PPD skin test, regardless of their BCG vaccination status and in accordance with already published studies [10]. Throughout the observation period of 58 weeks the PPD induced skin reaction increased to a maximum of 12 mm for the BCG vaccinated animals which was in the same range as reactions in the non-vaccinated animals (mean diameter of 10.5 mm). Skin test responses in the non-vaccinated animals were not measured after week 20 due to the development of severe clinical disease in this group.

Four weeks post infection E6/C10 induced a positive skin reaction in all non-vaccinated animals tested (n = 7) with a mean of 12 mm and the animals remained positive to E6/C10 throughout the observation period of 20 weeks until the animals were euthanized. In contrast to this, only 1 out of 7 in the BCG vaccinated group was positive to E6/C10 four weeks after infection whereas all animals (7 out of 7) were PPD positive at the same time. The mean E6/C10 skin test response increased in the BCG vaccinated group during the remainder of the experiment from a mean of 5 mm at week four, reaching a mean of 14 mm at week 52 (figure 1B). The percentage of E6/C10 positive animals in the BCG vaccinated group also increased as the disease developed. Up to week 12 post-infection, only 29% (2 out of 7) of the BCG vaccinated animals were positive (>5mm) to E6/C10. At week 20 after infection, the time point where animals in the BCG vaccinated group started succumbing to the disease, E6/C10 induced a positive skin test response in 71% (6 out of 7) of the tested animals and when the experiment was terminated at week 58, all of the surviving five animals were positive by the E6/C10 skin test. Neither PPD nor E6/C10 induced a skin test response in the non-infected control animals (data not shown).

### Correlation between skin test reaction and survival time post skin test

Our experiment showed that non-immunized animals developed a positive E6/C10 skin test response soon after infection and remained skin test positive until the animals were euthanized due to progressive disease. In contrast, the BCG-vaccinated group of animals initially controlled the disease and had a markedly lower E6/C10 skin test reaction size the first 12 weeks after infection. However, after 20 weeks of infection, this group of animals also developed a positive E6/C10 skin test reaction and the mean of the skin reaction continued to increase up to the point where the animals were euthanized. We therefore investigated if a correlation existed between the size of the skin test reaction and survival time post skin test for the individual animals. This analysis was based on the first skin test measurement for each animal and we pooled data for the two groups to get a larger sample size for the total analysis (figure 2A). This analysis demonstrated a clear negative correlation between the magnitude of the skin test response and survival time. This was confirmed by a Spearman's rank correlation test; r = −0.6 and P<0.0001. Also a statistical analysis at a fixed test time point, i.e. the Spearman's correlation test of data from only week 4, 8 or 12 post infection gave a statistically significant correlation between skin test size and survival time (week 4: *r* = −0.59, P = 0.0046 and 95 % confidence interval: −0.82, −0.2) but with larger confidence interval compared to the analysis with all 44 animals included. No correlation between the magnitude of the skin test response and survival time after infection was found using PPD as an antigen; Spearman *r* = 0.27 and P = 0.095, figure 2B.

## Discussion

In developed countries, targeted tuberculin testing for LTBI has long been a strategic component of TB control and individuals thought to be at high risk of developing TB are offered treatment [15]. This includes recent contacts and in particular those with clinical conditions that are associated with an increased risk of progression of LTBI to active TB (e.g HIV infection). However, in TB endemic regions with widespread transmission it is much more complicated to identify recent exposure and due to the high prevalence of LTBI (up to 80% of adults [16]) the approach of offering treatment to all infected individuals is not feasible. Our study suggests that a more targeted approach may be possible and that the size of an E6/C10 induced skin test response may identify incipient disease before onset of clinical TB. This concept is not unprecedented–TB-endemic countries routinely use a higher cut-off for positivity in the TST than low endemic countries based on the recognition that the magnitude of the response correlates with relative risk, albeit poorly.

We used the guinea pig model for a proof of concept study and took advantage of the fact that in outbred Dunkin Hartley Guinea pigs, BCG vaccination resulted in a significantly delayed onset of symptoms and prolonged survival after infection. Importantly, as these animals came from a genetically variable outbred colony, both vaccinated and unvaccinated cohorts differed markedly and had a wide range in survival time after infection, as documented previously in numerous studies [13], [9]. In the present study, this individual variation allowed the investigation of the relationship between clinical outcome and skin test size at the level of the individual animal. We demonstrated a clear association between the magnitude of the skin test response to E6/C10 and the subsequent development of clinical disease and a large skin test response soon after infection was found to correlate with a shorter period before onset of disease. The difference in response patterns for the vaccinated and the naïve animals was particularly prominent in the early phase after infection. Interestingly, this correlation was not related to the skin test per se, as the PPD skin test response was high throughout the infection period in all animals (p = 0.095) in line with what has already been published [5], [17].

Our study was limited by the risk of sensitization by multiple skin tests and it was therefore not possible to follow the development of the skin test response on an individual level for the animals. In addition, the study did not have statistically power to define a skin test cut off for incipient disease.

Our finding extends a number of recent reports from animal models on the use of ESAT-6 induced IFN-γ as an immunological marker that predicts disease progression. In Dietrich *et al.* [6] a number of subunit vaccines were tested for efficacy against infection with *M. tuberculosis* and a striking correlation was observed between the magnitude of a post challenge ESAT-6 IFN-γ response and the subsequent outcome of disease. In a study by Vordermeier et al [5], cattle were experimentally infected with *M. bovis* and the ESAT-6 specific IFN-γ responses were followed until the animals were euthanized. Again, disease severity, measured by pathology examination and bacterial count in the infected organs, corresponded to the magnitude of the ESAT-6 specific response.

Although data from humans addressing this correlation are still limited, one small study clearly demonstrated that the magnitude of the ESAT-6 specific IFN-γ response in recently exposed healthy contacts correlated with disease status one year later [7]. Consistent with this, in a large study involving 1052 healthy household contacts from the Gambia it was found that the number of cells producing IFN-γ in response to PPD and ESAT-6 correlated with the level of exposure to *M. tuberculosis*, even though this was just a trend for ESAT-6 (p = 0.08) [18]. Importantly, no such relationship across the exposure gradient was found for the TST. It might seem paradoxical that immune responses to ESAT-6, an antigen with documented vaccine efficacy in animal models [13] can serve as a marker for failing immunity. In most cases, an early induction (before the infection is established) of a vaccine driven ESAT-6 immune response leads to the suppression of infection and the subsequent down regulation of the immune response. In some individuals, however, the bacteria survive by manipulating the host's immune response [19]. As ESAT-6 is secreted by the replicating bacteria [20], [21], the infection driven immune responses to this antigen in susceptible individuals therefore correlates with the progression of the infection.

It is estimated that on average, each new TB case can infect up to 10 susceptible contacts before diagnosis [22] and with 8.9 million TB cases in 2004, an enormous number of new infections occurs each year [23]. 80 % of all cases are found in the 22 highest-burden countries in the developing world and due to the continuous reactivation of the disease in latently infected individuals, it is an extremely difficult task to break the cycle of infection. It would demand huge investments to implement the IGRAs in areas where the health care systems are generally of a poor standard and the resources limited. In this context, a novel skin test may have unique advantages compared to IGRAs. It is completely independent of laboratory facilities and can therefore be performed and evaluated on-site even in populations where blood sampling remains problematic for practical reasons (for example in young children) or on religious grounds. Furthermore, due to the existing widespread use of the TST, the health care personnel in TB endemic areas are in most cases already qualified and experienced in performing and evaluating skin tests.

We have recently completed a double-blind randomized Phase I study comparing ESAT-6 to the TST in healthy volunteers and previously treated TB patients [24]. ESAT-6 was found to be both safe and highly biologically active but interestingly no statistically significant association existed between the *in-vitro* IFN-γ response and the ESAT-6 skin test responses. The reason for this difference may reflect difference in the kinetics, homing potential or immune cell subsets involved in the biological functions measured in the two tests.

We are currently planning clinical studies to define the skin test dose and cut off value giving the highest predictive value for subsequent disease. In the light of the results in the present study, it is our hope that it may be possible to establish one cut off value for infection and a higher cut off value for incipient disease. In addition, it remains to be investigated if skin test size can be used as a single criterion or whether serial testing and conversion is necessary for prognosis of TB disease, as recently discussed elsewhere [25]. This would resemble the use of TST conversion to identify persons of increased risk-such as, for example, the periodic screening of health care workers which although far from ideal remains a useful component of TB control programmes in many countries.

The development of a test that can predict the 5–10% of a latently infected population at the highest risk of developing TB would change current practice dramatically and allow the prioritization of treatment within the population. This would lead to the best use of the scarce resources available and offer us the possibility of breaking the cycle of transmission. Short of a new vaccine, which is active against latent TB, such an approach offers us the best hope of controlling the global problem of LTBI.
